# Availability of adolescent mental, sexual and reproductive health services (ASRH) and technical efficiency of ASRH service delivery in Niger

**DOI:** 10.1186/s12913-026-15014-y

**Published:** 2026-06-30

**Authors:** Nassirou Ibrahim, Roxane Borgès Da Silva, Saidou Oumarou, Ama Pokuaa Fenny, Jacob Novignon, Michel Adurayi Amenah, Aïssa Diarra, Ludovic Deo Gracias Tapsoba, Annick Gladzah, Irene Akua Agyepong, Tim Ensor

**Affiliations:** 1 Laboratoire d’études et de recherches sur les dynamiques sociales et le développement local (LASDEL), Niamey, Niger; 2 École de santé publique, Université de Montréal, Montréal, Canada; 3 Institute of Statistical Social and Economic Research, University of Ghana, Accra, Ghana; 4 Ghana College of Physicians and Surgeons, Accra, Ghana; 5 Centre for Social Policy Studies, University of Ghana, Accra, Ghana; 6 Dodowa Health Research Centre, Accra, Ghana; 7 Laboratoire de santé publique, Université Joseph KI ZERBO, Ouagadougou, Burkina Faso; 8 University of Leeds, Leeds, UK

**Keywords:** Sexual and reproductive health services, Mental health, Resource utilization, Technical efficiency, Adolescents, Niger

## Abstract

**Background:**

In developing countries, many organizations are attempting to implement interventions to improve adolescent mental, sexual, and reproductive health (ASRH). A key challenge in these efforts is the efficient utilization of available resources. However, there is a considerable lack of efficiency studies, particularly in high resource-constrained settings such as Niger. Addressing this gap, the present study assessed the delivery of adolescent mental, sexual, and reproductive health services in Niger, while evaluating the technical efficiency and its determinants within public health facilities providing adolescent sexual and reproductive health services.

**Methods:**

A cross-sectional survey was conducted at 160 rural and urban health facilities in Niamey and Maradi, from May 31 to June 24, 2022. Descriptive frequency analysis assessed the availability of mental and sexual/reproductive health services. Stochastic frontier analysis (translog function) measured technical efficiency, while fractional logit regression identified influencing factors.

**Results:**

Over 89% of surveyed health facilities in Maradi and Niamey reported providing sexual and reproductive health services to adolescents. Conversely, the availability of mental health services was limited, with only 10% of facilities offering such support in these regions. Stochastic estimation revealed an average technical efficiency score of 45.2% (95%CI: 0.410–0.49). Notably, higher-than-average efficiency scores were observed among facilities managed by male personnel.

**Conclusion:**

The findings highlight significant gaps in the provision of mental health services, underscoring the need for targeted interventions. Training health personnel to effectively manage adolescents with mental health disorders is recommended. The comparatively low technical efficiency score suggests room for improvement; establishing well-equipped and functional pharmacies with relevant sexual and reproductive health products may contribute to enhanced performance in health service delivery.

**Supplementary information:**

The online version contains supplementary material available at https://doi.org/10.1186/s12913-026-15014-y.

## Background

Adolescence, defined as the period from 10 to 19 years of age, is a critical developmental stage characterized by rapid physical, cognitive, social, emotional, and sexual changes [1–3]. In West and Central Africa, adolescents represented 23% of the population in 2019, the highest regional proportion globally, and this share is projected to increase by 32% between 2019 and 2030 [4–6]. This demographic trend underscores the growing need for responsive policies and services tailored to adolescents, particularly in the areas of mental health and sexual and reproductive health.

Mental health disorders are a major concern during adolescence. A meta-analysis estimated the global prevalence of mental disorders among children and adolescents at 13.4% (95% CI: 11.3–15.9) [7]. However, most available studies were conducted in high-resource settings, with very limited evidence from sub-Saharan Africa and almost none from Niger. Because the expression of psychological distress and the interpretation of symptoms are strongly shaped by social and cultural context [8], this evidence gap limits the development of appropriate screening tools, needs assessments, policies, and interventions for adolescents in African settings [9].

Adolescent sexual and reproductive health remains another major public health challenge. Although adolescent pregnancy and childbirth have declined in some contexts as school enrolment, contraceptive demand, and age at first marriage increase, levels remain high in several parts of West Africa. Globally, 11% of all births occur among girls and young women aged 15–19 years, most of whom live in low- and middle-income countries. Sub-Saharan Africa accounts for 4.5 million of the 14 million births recorded annually in this age group [10]. In addition, more than 4.4 million abortions occur each year among these young women, with approximately half taking place under unsafe conditions. In West Africa, the average adolescent fertility rate remains high, at 124 births per 1,000 live births [11].

In Niger, adolescent girls contribute approximately 16% of total fertility [12, 13]. Sexual debut occurs early, with a reported mean age at first intercourse of 15 years for girls and 20 years for boys, and more than one-third of adolescent girls have had at least one child before the age of 17. Adolescent maternal mortality represents 69% of total maternal mortality. Although knowledge of contraceptive methods is relatively high among adolescents (76% among boys and 82% among girls), actual use remains extremely low, with only 1.3% reporting the use of at least one method, compared with an already low national average of 8% [12, 13].

Adolescent sexual and reproductive health (ASRH) refers to the physical and emotional well-being of adolescents and young people, including their capacity to prevent unintended pregnancy, unsafe abortion, sexually transmitted infections (STIs), including HIV, and sexual violence or coercion [14, 15]. According to the Cairo Programme of Action, ASRH comprises five main components: maternal and newborn health, family planning, prevention of unsafe abortion, infection management, and promotion of sexual health [2, 3, 16]. Depending on national priorities and capacities, countries have implemented interventions covering one or more of these components, including family planning, pregnancy follow-up, prevention and treatment of STIs, HIV-related services, sexuality education, and prevention of unintended pregnancy [16].

In Niger, ASRH interventions have been supported by several international partners, including United Nations agencies, the World Bank, UNICEF, UNFPA, the Bill & Melinda Gates Foundation, research centres, and bilateral or non-governmental organizations [17]. Although these investments have contributed to improvements in service provision, adolescent-specific service availability remains insufficient. For example, in 2017, only 29% of health facilities in Niger provided family planning services to unmarried adolescents, compared with 54% in Ghana and 70% in Burkina Faso [1, 6, 18–20].

Recent studies indicate that public and private organizations implementing ASRH interventions face multiple constraints that affect both service production and utilization [1, 18, 21, 22]. Among these constraints, the efficient use of available resources is a recurring challenge intervention [18, 21, 22]. The delivery of health services requires a combination of technical, managerial, adaptive, and leadership capacities [23–28]. Nevertheless, only a limited number of studies have examined efficiency in the provision of ASRH services in low-resource settings, and available findings suggest that technical efficiency scores in adolescent health service delivery often range from 40% to 60% [29–31].

Efficiency is a multidimensional concept commonly described in terms of technical, allocative, and economic efficiency [32–38]. Technical efficiency refers to the ability of an organization to maximize service outputs from a given set of inputs. Allocative efficiency reflects the capacity to use inputs in optimal proportions based on their costs. Economic efficiency combines these two dimensions and refers to producing the maximum possible services at optimal cost [39, 40]. In practice, the analysis of allocative and economic efficiency requires reliable information on input costs and output prices, which is often difficult to obtain in low-income settings. Consequently, technical efficiency is frequently the most feasible dimension to assess in such contexts.

Given these challenges, generating evidence to improve adolescent health and well-being in Niger is essential. This study focuses on the regions of Maradi and Niamey and examines: (i) the provision of adolescent mental health services and adolescent sexual and reproductive health services; and (ii) the technical efficiency of health facilities in delivering ASRH services, as well as the determinants of that efficiency. The analysis of technical efficiency was limited to ASRH services because data on adolescent mental health services were insufficient: fewer than 10% of the visited health facilities reported offering such services, preventing a robust efficiency assessment in that domain.

## Methods

The study design was a cross-sectional survey of primary care facilities providing adolescent sexual and reproductive health services in the Niamey and Maradi regions of Niger.

### Data and sampling

A stratified, multistage sampling strategy was implemented to select primary care facilities. Initially, researchers chose two regions of Niger—Maradi and Niamey—based on their sociodemographic and health profiles. Maradi ranks among the regions with the lowest adolescent sexual and reproductive health indicators, whereas Niamey shows comparatively better statistics. For instance, teenage contraceptive use stands at 6% in Maradi and 9% in Niamey. The selection of these areas is further supported by their contrasting distributions of health facilities: 90% of Niamey’s centres are in urban settings, while 82% of Maradi’s operate in rural areas. This approach ensured both urban and rural populations were adequately represented in the sample.

In the second stage, five health districts were selected from each region. Since Niamey only has five districts, all of them were included. In Maradi, the Maradi health district was intentionally selected because it covers urban areas, while the other four districts—Madarounfa, Dakoro, Mayahi, and Tessaoua—were randomly selected from eight additional rural-urban districts using a Random Number Generator (RNG).

In the third stage, 160 health facilities: 100 in Maradi and 60 in Niamey were selected from a total of 184. The sample size was determined based on each region’s proportion of health facilities, the number of data collectors available, and the time allocated for the survey. Health facility selection began with stratification by region and district, followed by random sampling proportional to each district’s size (see Table 1). Nevertheless, a quota of seven private health facilities (five in Niamey and two in Maradi) was randomly selected to represent private facilities. In Niamey, one private facility was randomly chosen per district, while Maradi’s limited private options meant just two were randomly selected in Maradi city. The remaining 153 public facilities were assigned proportionally according to their presence within each district and then randomly chosen. All selections used the RNG application available on the Play Store.

**Table 1:** Sample size by district and region

Region	District	Number of health facilities available	Proportion (%)	Sample
Maradi	DS Dakoro	25	22	22
	DS Madarounfa	20	18	18
	DS Maradi Ville	17	15	15
	DS Mayahi	29	26	26
	DS Tessaoua	21	19	19
	**Total Maradi**	**112**	**100**	**100**
Niamey	DS Niamey I	19	26	16
	DS Niamey II	13	18	11
	DS Niamey III	11	15	9
	DS Niamey IV	16	22	13
	DS Niamey V	13	18	11
	**Total Niamey**	**72**	**100**	**60**
**Total**		**184**		**160**

Source: Authors using data from the AdoWA project

However, for the technical efficiency and regression analyses, only 105 observations were retained. This is because 18 health facilities reported that they did not offer sexual and reproductive health services and 37 health facilities did not have a complete information on both output and inputs to determine their technical efficiency score.

### Stochastic frontier analysis (SFA) model

A technical efficiency analysis and linear regression were conducted. The technical efficiency analysis produced scores quantifying the efficiency of health facilities. Linear regression was subsequently utilized to identify factors influencing these technical efficiency scores.

Nonparametric and parametric approaches are generally used to assess technical efficiency score [34, 40]. Data envelopment analysis (DEA) is a nonparametric approach that requires only input and output data. However, the limitation of DEA is that it does not consider the possible influence of measurement error and other noise in the data. Stochastic frontier analysis, which is a parametric approach, takes this limitation into account, which is why it has been favoured in this study [34]. The econometric formulation of this approach for cross-sectional data is given by:

1 $${Y_i} = f({X_i};\beta ).exp({\varepsilon _i})$$

With $${\varepsilon _i} = {v_i} + ( - {u_i})$$.

where${Y_i}$ is the output level (here, it is the number of sexual and reproductive health visits made by adolescents at the health facility level) for health facility i,$f({X_i};\beta )$ denotes the production function (Cobb‒Douglas or Translog) of the row vector of inputs ${X_i}$ (see Table 2), a vector β of parameters to be estimated,${v_i}$ is the error term, and$( - {u_i})$ is a nonnegative random variable associated with specific factors that cause the ith health facility to fall short of its maximum efficiency.

**Table 2:** Description of analysis variables

Variable names	Measurement	Stochastic frontier model	Fractional Logit regression model
**External/dependent variable**			
Ln (Number of sexual and reproductive health visits by adolescents in 2021) (Y)	Number of sexual and reproductive health visits by adolescents in 2021	Yes	No
Technical efficiency score	Score of technical efficiency of health facility	No	Yes
**Inputs/independent variables**			
Ln (Number of midwives working with adolescents) (X_1_)	Number of midwives working with adolescents	Yes	No
Ln (Number of nurses working with adolescents) (X_2_)	Number of nurses working with adolescents	Yes	No
Ln (Number of administrative staff working with adolescents) (X_3_)	Number of administrative staff working with adolescents	Yes	No
Ln (Average time spent on an adolescent patient) (X_4_)	This variable is the average of the time spent on average by a healthcare provider within the healthcare facility. It is an average derived from data recorded in the healthcare facilities’ consultation registers and appointment schedules, where available. It is expressed in minutes.	Yes	No
**Control variables**			
Place of residence	A dummy variable that takes the value of 1 if a facility is in an urban area and 0 otherwise.	No	Yes
Gender of health facility head	A dummy variable that takes the value of 1 if a facility is headed by a male and 0 if a female.	No	Yes
Percentage of adolescents in the covered area	The percentage of the Population of the catchment area of the health facility who are adolescent.	No	Yes
Number of vehicles in operation	Number of vehicles in operation.	No	Yes
Regular access to electricity	A dummy variable that takes the value of 1 if a facility has regular access to electricity and 0 otherwise.	No	Yes
Regular access to drinking water	A dummy variable that takes the value of 1 if a facility has regular access to drinking water and 0 otherwise.	No	Yes
Availability of a functional pharmacy	Dummy variable that took 1 if the health facility had a functional pharmacy and 0 if no.	No	Yes
Existence of at least one trained ASRH staff	A dummy variable that takes the value of 1 if a facility has the existence of at least one trained ASRH staff and 0 otherwise.	No	Yes
Existence of national ASRH guidelines	A dummy variable that takes the value of 1 if a facility has existence of national ASRH guidelines and 0 otherwise.	No	Yes

Source: Authors using data from the AdoWA project

When we consider a translog model, the relationship (1) becomes:

2 $$\eqalign{& {Y_i} = f({X_i};\beta ) \cr & = \exp \left( {{\beta _0} + \sum\limits_{i = 1}^N {{\beta _i}} \ln {x_i} + {1 \over 2}\sum\limits_{i = 1}^N {\sum\limits_{j = 1}^N {{\beta _{ij}}\ln {x_i}\ln {x_j}} } } \right) \cr & .exp({v_i}).exp( - {u_i}) \cr} $$

where ${X_i}$ is the vector of inputs (see Table 2), ${\beta _i}$ are the parameters for inputs ${X_i}$, and N is the number of inputs.

In addition, for the estimation of the function, we proceeded to a logarithmic transformation to obtain the linear function of the model that was estimated. Thus, the relationship (2) becomes:

3 $$\ln ({Y_i}) = {\beta _0} + \sum\limits_{i = 1}^N {{\beta _i}} \ln {x_i} + {1 \over 2}\sum\limits_{i = 1}^N {\sum\limits_{j = 1}^N {{\beta _{ij}}\ln {x_i}\ln {x_j}} } + ({v_i} - {u_i})$$

where ln is the natural logarithm. Parameter estimation of (3) is underpinned by distributional assumptions about the two error terms. The measure of technical efficiency scores for the ith health facility is denoted by $E{T_i}$.

4 $$E{T_i} = {{{Y_i}} \over {Y_i^*}} = {{f({X_i};\beta ).exp({v_i} - {u_i})} \over {f({X_i};\beta ).exp({v_i})}} = \exp ( - {u_i})$$

A value near to 0 indicates that the facility is technically less efficient in using available resources (inputs) to provide adolescent sexual and reproductive health services. In contrast, a value close to 1 indicates that the facility is technically more efficient.

Output orientation keeps resource use steady and looks at ways to increase service delivery, while input orientation maintains output levels and seeks to lower resource consumption. For this study, we focused on output orientation because the health system usually manages outputs directly and operates using only the resources provided. Also, when it comes to health care, expanding outputs tends to be a more straightforward approach.

### Factional regression model

#### Model specification

Fractional regression is like ologit regression in the sense that it can be used to model a variable that takes values within a bounded range. A key difference is that the dependent variable can be measured as continuous and does not need to be converted to categories. As the efficiency score is bounded in the [0,1] interval, the use of the ordinary least squares (OLS), the Tobit regression, or the transformed normal logistic model (the log-odds ratio of the dependent variable) in such cases is inefficient, as their error distributions will be heteroskedastic. In this situation, Papke and Wooldridge [41] proposed a fractional response model (FRM), which is an extension of generalized linear models (GLMs). The fractional response model is a nonlinear model estimated using the quasimximum likelihood estimation (QMLE) method. The QMLE is asymptotically more efficient and more consistent than either OLS or Tobit methods [42, 43].

The fractional logit model is used to analyse the factors influencing the technical efficiency score, as in Papke and Wooldridge [41]. The model is as follows:

$$E{\rm{(}}{{\rm{Y}}_i}{\rm{| }}{X_i}) = G\left( {{X_i}\beta } \right),i = 1, \ldots ,N,$$

where $0{\rm{ }} \le {\rm{ }}{{\rm{Y}}_i} \le 1$ denotes the dependent variable, which in our case represents the efficiency score, (the 1× k vector) ${X_i}$ represents the explanatory variables (see Table 2) of observation i, β is a vector of unknown parameters, and $G{\rm{ }}\left( . \right)$ is a distribution function that follows a logistic distribution function defined as:$G({{\rm{X}}_i},{\rm{\beta }}) = {{\exp \left( {{{\rm{X}}_i}{\rm{\beta }}} \right)} \over {1 + \exp \left( {{{\rm{X}}_i}{\rm{\beta }}} \right)}}$

The quasimaximum likelihood estimation (QMLE) method is used to estimate unknown parameters based on the Bernoulli log-likelihood function, which is defined as:

$${L_i}\left( \beta \right) = {{\rm{Y}}_i}*ln\left[ {G\left( {{X_i}\beta } \right)} \right] + \left( {1 - {{\rm{Y}}_i}} \right){\rm{ln}}\left[ {1 - G\left( {{X_i}\beta } \right)} \right]$$

#### Validity test of the model

A likelihood ratio (LR) test was performed to choose the form of the function used for the translog model in the stochastic frontier analysis. Then, a chi-square test was performed to determine whether the presence of inefficiencies was justified.

For fractional logit regression, a nullity test of the coefficients was carried out to guarantee the validity of the model. We also performed a specification test using a fitted model with residuals and squared residuals. The results of this test showed that the coefficient associated with the square of the residuals was statistically insignificant. Consequently, the unadjusted residual model was very well specified.

### Variables and measurement

*Output:* the number of sexual and reproductive health visits made by adolescents during 2021 is the output of production function in the technical efficiency (see Table 2). This variable was logarithmic transformed to obtain a linear form while it does not capture outcomes beyond the health facility, it presents an opportunity to compare health facilities as it captures the care process under the control of the health facilities. The choice of “number of adolescent sexual and reproductive health consultations” as output was initially based on the literature. Indeed, several studies had also used the number of consultations or visits recorded in health facilities as an output or dependent variable to determine the technical efficiency scores of health facilities, particularly in developing countries where data quality is not always guaranteed [44–46]. Otherwise, indicators relating to patient outcomes or satisfaction could be used as outputs, but information on these indicators was not available at the health facilities visited.

*Inputs:* Four inputs were used in the estimation of the production function. These include number of midwives working with adolescents, number of nurses working with adolescents, number of administrative staff working with adolescents, and average time spent on an adolescent patient (see Table 2). As with the output, all input variables were logarithmically transformed to obtain their linear form. Similarly, the choice of input variables was based first on literature and then on the availability of information enabling many structures with complete information on inputs and outputs to be identified to conduct relevant technical efficiency analysis [29–31]. Particularly the inclusion of time as an input is theoretically grounded in health production theory because it represents a critical resource in health care delivery, reflects service intensity and human resource utilization, capture opportunity costs, and aligns with the specific characteristics of adolescent-friendly sexual and reproductive health services [30].

To complete the model, other variables with a potential influence on the technical efficiency of health facilities were used in the model. These variables are sociodemographic characteristics of health facilities and contextual factors (see Table 2).

## Results

### Availability of adolescent sexual and reproductive health (ASRH) and adolescent mental health (AMH) services

#### Availability of *ASRH services but almost no availability of AMH services*

**Fig. 1:**
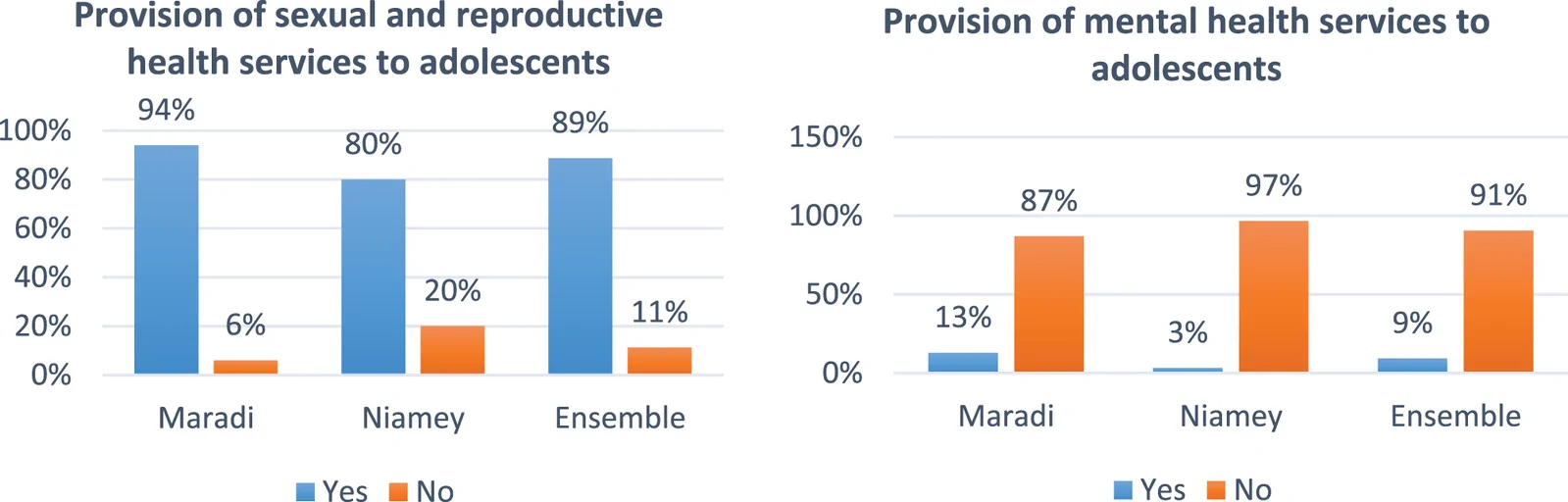
Distribution (in %) of health facilities by type of service offered (source: authors using data from the AdoWA project)

Since the International Conference on Population and Development (ICPD) in 1994, Niger has implemented important interventions to improve adolescent sexual and reproductive health. This has led to improved availability of these services. Indeed, several health facilities offer adolescent sexual and reproductive health services in the two target regions. As shown in Fig. 1, approximately 89% of the health facilities surveyed reported offering SRH services to adolescents. This proportion is greater in Maradi (94%) than in Niamey (80%), but the difference is not significant. These results reflect the effects of several programs implemented by the government of Niger since the 2000s to make ASRH services available and accessible.

On the other hand, the supply of mental health services is very mixed. In both regions, barely 9% of the health facilities surveyed offer these services to adolescents (Fig. 1). Regional disparities also emerge. In Maradi, the proportion was 13%, while in Niamey, it was only 3%.

#### National guidelines exist for ASRH but are almost non-existent for AMH

**Fig. 2:**
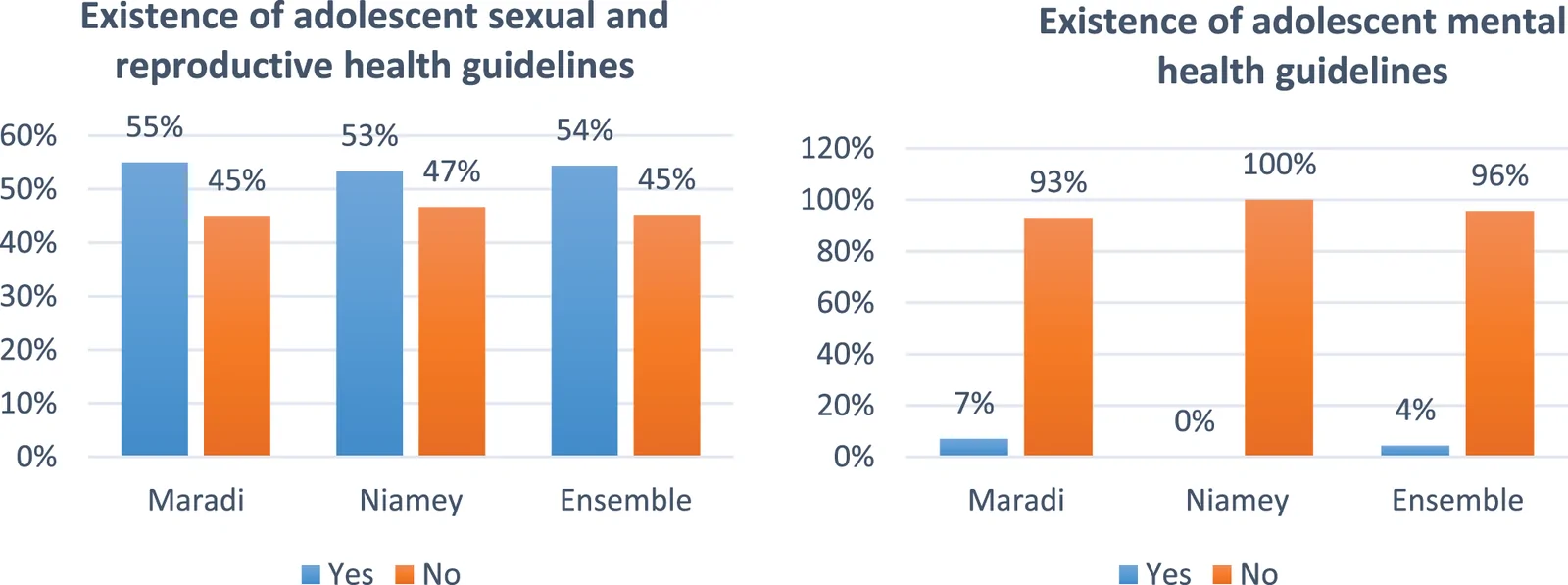
Distribution of surveyed health facilities (in %) by region according to the existence of national guidelines for the provision of SRH and SM services to adolescents (source: authors using data from the AdoWA project)

Providing sexual and reproductive health (SRH) or mental health (MH) services to adolescents remains a significant challenge for numerous facilities. As illustrated in Fig. 2, 54% of surveyed facilities reported the existence of national guidelines for adolescent SRH services, with the proportion slightly higher in Maradi (55%) and lower in Niamey (53%).

In contrast, only 4% of health facilities across all target regions indicated having adolescent MH guidelines. Notably, no facilities surveyed in Niamey reported the presence of national directives for adolescent mental health services, whereas in Maradi, 7% of facilities did identify such guidelines.

#### Many health facilities visited have at least one provider trained in ASRH services, but almost all of them do not have in AMH services

**Fig. 3:**
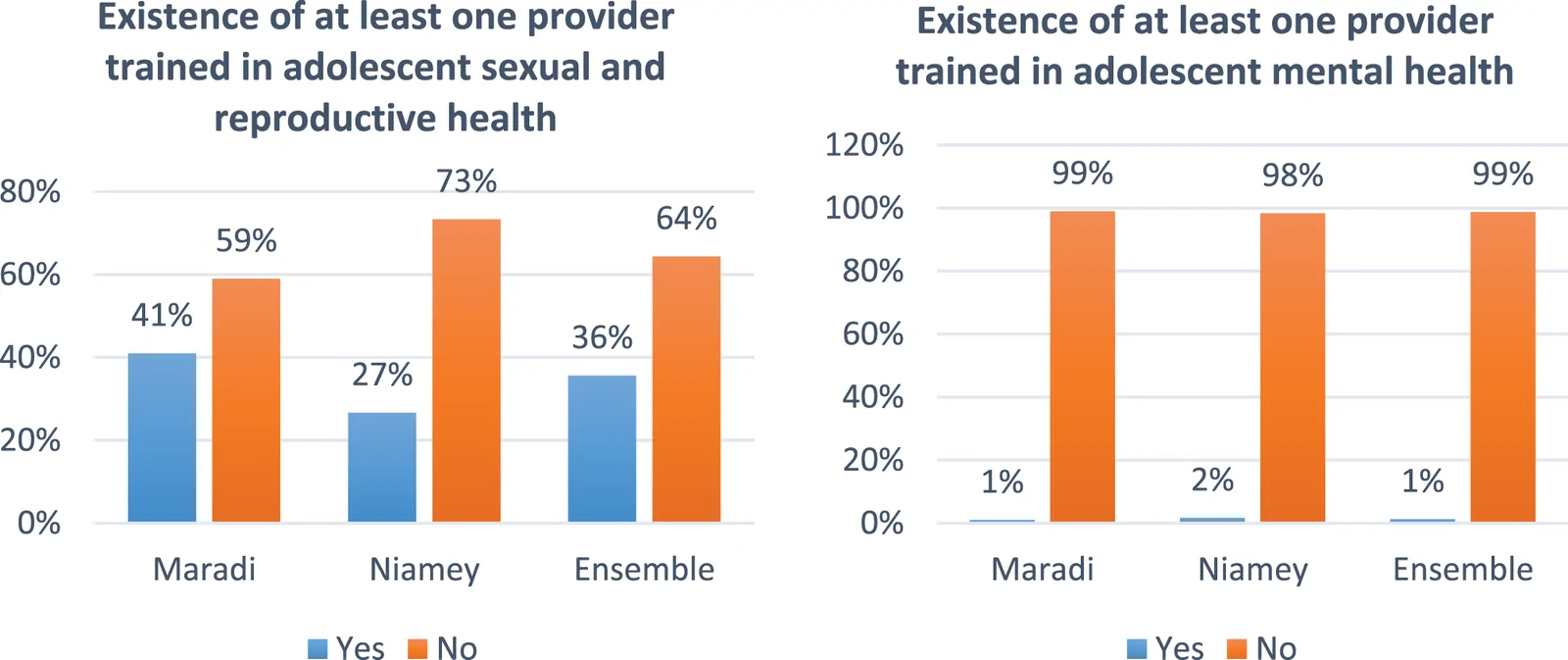
Distribution of surveyed health facilities (in %) by region according to the existence of at least one provider trained in adolescent-specific services (source: authors using data from the AdoWA project)

Health standards require that adolescent sexual and reproductive health services be delivered by professionals trained specifically in adolescent care. In 2006, Niger launched the Adolescent Friendly Program with backing from organizations like UNFPA, training providers in youth-focused services. Although the program ended in 2012, the government continued efforts to train health care staff on adolescent issues. According to statistics, 40% of surveyed health facilities in Maradi and Zinder have staff trained in sexual and reproductive health for adolescents (see Fig. 3). In Niamey, this figure is 27%, while in Maradi it is 41%. These numbers show there is still significant progress needed to ensure every health facility has at least one provider skilled in adolescent SRH questionnaires. Differences across regions may be due to greater NGO support in rural areas, as most NGOs focus more on regional locations than the capital, Niamey.

The situation regarding mental health is even more concerning. Out of 100 health facilities surveyed, very few had personnel trained in mental health care, highlighting a lack of focus on this area. Despite this, a significant number of adolescents are affected by psychological illnesses and disorders. Factors such as drugs and narcotics use, rape, domestic violence, and forced or early marriages are contributing to growing risks. A global meta-analysis estimated that 13.4% (95%CI: 11.3–15.9) of children and adolescents worldwide suffer from mental disorders. Unfortunately, Niger does not have available statistics on this issue.

### Descriptive statistics

Table 3 presents the descriptive statistics for the variables employed in calculating technical efficiency scores, as well as in analysing the factors that influence the technical efficiency of health facilities.

**Table 3:** Descriptive statistics

Variable	Obs.	Average	95%Confidence interval (CI)	
ln (Number of sexual and reproductive health visits by adolescents in 2021) (Y)	105	6.061	(5.749	6.372)
ln (Number of midwives working with adolescents) (X_1_)	105	0.452	(0.342	0.562)
ln (Number of nurses working with adolescents) (X_2_)	105	1.061	(0.923	1.198)
ln (Number of administrative staff working with adolescents) (X_3_)	105	3.194	(3.057	3.330)
ln (Average time spent on an adolescent patient) (X_4_)	105	0.026	(−0.005	0.058)
Place of residence	105	0.305	(0.215	0.394)
Management authority	105	0.933	(0.885	0.982)
Gender of health facility head	105	0.752	(0.668	0.836)
Percentage of adolescents in the covered area	105	27.933	(24.842	31.02)
Number of vehicles in operation	105	0.390	(0.285	0.496)
Regular access to electricity	105	0.514	(0.417	0.611)
Regular access to drinking water	105	1.181	(1.106	1.256)
Availability of a functional pharmacy	105	0.762	(0.679	0.845)
Existence of at least one trained ASRH staff	105	0.438	(0.342	0.535)
Existence of national ASRH guidelines	105	0.629	(0.535	0.723)

### Technical efficiency of health facilities in providing ASRH services

#### Estimation of the stochastic frontier model

Table 4 shows that both the Cobb-Douglas and translog models are statistically significant. However, the nullity test ${\beta _{ij}}$ results indicate that the null hypothesis cannot be accepted. Among the two, the translog model performs better than the Cobb–Douglas model, so it was chosen to compute the technical efficiency score. Additionally, the probability tied to the Wald chi statistic is significant at the 5% level, and the *p*-values for Usigma and Vsigma are also below 5%. This reveals technical inefficiencies in health facilities concerning the number of sexual and reproductive health consultations offered to adolescents in 2021.

**Table 4:** Stochastic frontier regression of the number of adolescent sexual and reproductive health visits

Variables	Cobb Douglas model	Translog model
Frontier		
X_1_	−0.063	−0.505
	(−0.23)	(−0.24)
X_2_	0.167	1.656
	(0.71)	(1.20)
X_3_	0.274	2.212
	(1.17)	(1.40)
X_4_	−2.038***	−6.171***
	(−2.97)	(−5.09)
X_1_^2^		0.421
		(0.56)
X_2_^2^		0.798**
		(2.13)
X_3_^2^		−0.230
		(−0.75)
X_4_^2^		3.550**
		(2.25)
X_1_*X_2_		−0.262
		(−0.45)
X_1_*X_3_		0.0766
		(0.17)
X_1_*X_4_		−0.618
		(−0.66)
X_2_*X_3_		−0.837**
		(−2.02)
X_2_*X_4_		0
		(.)
X_3_*X_4_		0
		(.)
_cons	6.382***	2.710
	(7.29)	(1.28)
sigma_u	1.291***	1.219***
	(4.98)	(4.04)
sigma_v	0.870***	0.787***
	(4.75)	(4.28)
Lambda	1.484***	1.55***
	(3.62)	(3.34)
**Number of obs.**	105	105
**Wald chi2(7)**	16.37	597.22
**Prob > chi2**	0.003	0.000

*Note: t* statistics in parentheses * *p* < 0.10, ** *p* < 0.05, *** *p* < 0.01

The findings indicate that more time spent by health facilities with adolescents is associated with a reduction in the number of adolescents seeking consultations on sexual and reproductive health. As a result, the overall volume of such consultations remains limited.

On the other hand, the current number of midwives has no significant influence on the number of adolescent SRH consultations. However, when squared, we see an increase in the number of adolescent SRH consultations.

#### Technical efficiency scores of health facilities

The efficiency score assesses the ability of facilities to use available inputs or resources to deliver the maximum number of services to adolescents. According to Table 5, the average technical efficiency score across surveyed health facilities was 45.2% (95%CI: 0.410 0.494). This means, given current input levels, the facility could produce 1/0.452 ≈ 2.212 times of the current output if it were fully efficient on the frontier. Technical efficiency score is higher in the Maradi region (46%) compared to the Niamey region (43%). Furthermore, health facilities managed by men exhibit greater technical efficiency (47%) than those managed by women (45%). It is important to emphasize that this analysis offers insight into the variability of technical efficiency scores among different categories of health facilities but does not explicitly identify their underlying determinants.

**Table 5:** Efficiency scores by facility characteristics

Variable/category	N	Average	Standard deviation	Min	Max	95%CI
**Gender of health facility head**						
Woman	26	0.407	0.210	0.017	0.790	(0.323 0.492)
Male	79	0.467	0.218	0.009	0.765	(0.418 0.516)
**Place of residence**						
Rural	73	0.450	0.217	0.009	0.765	(0.340 0.501)
Urban	32	0.457	0.218	0.010	0.790	(0.378 0.536)
**Management authority**						
Government	98	0.459	0.215	0.009	0.790	(0.416 0.503)
Private	7	0.353	0.218	0.0343	0.697	(0.151 0.554)
**Region**						
Maradi	87	0.457	0.217	0.009	0.779	(0.410 0.503)
Niamey	18	0.430	0.219	0.010	0.790	(0.321 0.539)
**overall**	**105**	**0.452**	**0.216**	**0.009**	**0.790**	(0.410 0.494)

### Determinants of technical efficiency in health facilities

The estimated model is significant at the 5% level, and the pseudo-R2 value is 5%. This means that there is at least one variable in the estimated model that explains the technical efficiency scores and that the model is predictive at 5%.

The results indicate that many factors are associated with the technical efficiency scores. However, these factors did not operate in the same way. For example, health facilities with a lower proportion of adolescents in the coverage population are more technically efficient than those with a high proportion of adolescents in the covered population. Also, a possible positive association between male facility leadership and technical efficiency; however, this relationship was not statistically significant at the 5% level but at 10%. Health facilities with a functional pharmacy are more technically efficient at providing adolescent services than are those without a functional pharmacy. As shown in Table 6, health facilities with a functional pharmacy are twice as technically efficient as those without a functional pharmacy. The provision of adolescent sexual and reproductive health services most often requires specific skills that require training. The results shown Health facilities with at least one trained provider for adolescent sexual and reproductive health services are 1.5 times more technically efficient than those without a trained provider.

**Table 6:** Estimation of parameters using fractional logit regression

Variables/category	Odd ratio	St. error	p > t	95%CI	
Place of residence					
*Rural*	Ref.				
*Urban*	1.450	0.395	0.173	(0.850	2.475)
Gender of health facility head					
*Woman*	Ref.				
*Man*	1.511*	0.348	0.073	(0.962	2.374)
Proportion of adolescents in the covered area	0.990**	0.005	0.049	(0.981	1.000)
Managing Authority					
*Private*	Ref.				
*Government*	3.982***	1.486	0.000	(1.916	8.275)
Regular access to electricity					
*No*	Ref.				
*Yes*	0.903	0.201	0.648	(0.584	1.398)
Regular access to drinking water					
*No*	Ref.				
*Yes*	0.621*	0.155	0.056	(0.382	1.012)
Availability of a functional pharmacy					
*No*	Ref.				
*Yes*	1.998***	0.381	0.000	(1.375	2.904)
Existence of at least one trained ASRH staff					
*No*	Ref.				
*Yes*	1.531**	0.272	0.016	(1.081	2.168)
Existence of national ASRH guidelines					
*No*	Ref.				
*Yes*	0.863	0.151	0.399	(0.613	1.215)
Number of vehicles in operation	0.877	0.080	0.149	(0.733	1.048)
Constant	0.189***	0.107	0.003	(0.062	0.575)
**Observation**	105				
**Wald chi2(10)**	56.930				
**Prob > F**	0.000				
**Log likelihood**	−91.965				
**Pseudo R2**	0.048				

*Note:* * *p* < 0.10, ** *p* < 0.05, *** *p* < 0.01. CI: confidence interval

## Discussion

The findings indicate that access to mental health services remains severely constrained in the Maradi and Niamey regions. Only 9% of surveyed facilities reported offering such services. The extremely limited availability of mental health services and national mental health guidelines highlights persistent gaps in the integration of mental healthcare into primary and adolescent health services. These findings are particularly relevant within the framework of the WHO Mental Health Gap Action Programme (mhGAP), which promotes the decentralization and integration of mental healthcare into primary health systems in low-resource settings [47–50]. This result also underscores challenges in achieving Sustainable Development Goal 3.4, which explicitly calls for the promotion of mental health and well-being [51, 52]. Strengthening guideline dissemination, workforce training, and integration of mental health into adolescent SRH platforms may therefore represent important priorities for health system strengthening in Niger.

The findings also show that more than 89% of the health facilities surveyed in the Maradi and Niamey regions offer adolescent sexual and reproductive health (ASRH) services. Since the 1994 International Conference on Population and Development, many ASRH interventions have been implemented [1, 32]. For example, following countries such as Burkina Faso, Ghana, Senegal and other developing countries, Niger created the Youth Initiative for Adolescent and Youth Sexual and Reproductive Health in 2000. Under this initiative, “youth and adolescent friendly centres” were created in several regions of Niger to promote adolescent sexual and reproductive health. A few years later (i.e., in 2005), counselling centres were opened in all regions of Niger under the direction of the Ministry of Youth and with support from the UNFPA. These centres provide recreational activities and counselling for adolescents, including on sexual and reproductive health issues [1, 32, 33]. To further strengthen this service, the government introduced a program called the “Adolescent Friendly Program” during the period 2006–2012, in which approximately 40 health facilities were labelled adolescent and youth-friendly health facilities. Providers in these facilities were trained on the sexual and reproductive health needs of adolescents and the provision of services to this segment of the population [32]. The experience of this program has encouraged the government to provide ad hoc training to basic health facility providers on the provision of specific services to adolescents.

These different interventions have been accompanied by several funding sources, primarily from Niger’s technical and financial partners. These partners include United Nations agencies such as the World Bank, UNICEF and UNFPA, foundations such as the Bill & Melinda Gates Foundation, foreign governments directly or through institutions and agencies such as the Spanish Cooperation, the French Development Agency, Pathfinder and the International Planned Parenthood Federation. The State remains the second-largest source of funding. As a result of these efforts, the supply of ASRH services has improved, as indicated by the results of this study. However, despite the resources mobilized to implement ASRH interventions, indicator levels remain very critical [32]. This raises questions about the implementation of interventions.

The use of resources in the implementation of ASRH interventions is one of the factors that hinders the effectiveness of policies and programs aimed at improving the health and well-being of adolescents in developing countries [1, 18]. While some organizations can make efficient use of the resources mobilized, others struggle to do so. According to the results of this work, the technical efficiency scores of the health facilities that offer ASRH services are very low. The average score is 45% in the regions (Maradi and Niamey) targeted by this study. However, it is greater in the Maradi region (46%) than in the Niamey region (43%). Also, a possible positive association between male facility leadership and technical efficiency; however, this relationship was not statistically significant at the 5% level but at 10%. These findings are broadly consistent with the wider literature on health facility efficiency in low- and middle-income settings, which shows substantial variation in technical efficiency across facilities and highlights persistent methodological and contextual challenges in measuring performance. In addition to prior studies from Ghana and Niger [29–31], broader reviews by Hollingsworth and by Hussey et al. have shown that efficiency estimates are highly sensitive to the specification of inputs and outputs, the analytical approach used, and whether quality of care is incorporated into the analysis [35, 53]. Likewise, Hafidz et al. have emphasized that, in low- and middle-income countries, most efficiency studies rely on heterogeneous methods and often identify both supply-side and demand-side constraints as key determinants of performance [44]. This suggests that the relatively low scores observed should be interpreted not only as evidence of underperformance, but also within a broader body of work showing that inefficiency in low-resource settings is often shaped by structural constraints, service organization, and delivery arrangements. In addition, evidence syntheses on service integration in low- and middle-income countries suggest that better coordination and integration of primary health services may improve delivery processes and service utilization, although the effects depend heavily on implementation context [54, 55].

Fractional logit regression analysis indicates multiple factors associated with facility-level technical efficiency. These include the gender of the facility head, the proportion of adolescents within the catchment population, the presence of a functioning pharmacy, and the availability of at least one trained ASRH provider. Health facilities with trained ASRH staff demonstrate greater technical efficiency compared to those without. This finding is consistent with research by Jacob et al. in Ghana and Ibrahim et al. in Niger, which suggests that the specialized skills and professionalism required for effective ASRH service delivery—particularly regarding safety, confidentiality, and trust—are critical [18, 29, 30, 56]. Adolescents seeking safe and confidential services tend to select facilities upholding these values, which, due to increased demand, promotes optimal resource management for this population group.

The availability of a pharmacy within health facilities is significantly associated with higher technical efficiency (OR = 1.998, 95%CI: 1.375–2.904). This finding highlights the importance of pharmaceutical service readiness as a key determinant of health system performance in low-resource settings. Indeed, the availability of medicines is a cornerstone of effective service provision, influencing treatment adherence, clinical outcomes, and patient satisfaction. Health facilities with functional pharmacies are more likely to ensure continuity of care by providing immediate access to prescribed drugs, thereby increasing the volume of completed treatments. In efficiency terms, this results in higher outputs for a given level of input, which directly improves technical efficiency. Previous studies have consistently shown that medicine availability is strongly associated with service readiness and quality of care in low- and middle-income countries [57, 58].

The presence of a pharmacy may enhance the utilization of health services. Demand for health care is highly sensitive to the perceived availability of essential drugs at the point of care. Facilities lacking medicines often experience lower attendance rates and reduced trust among patients. Conversely, facilities with reliable pharmaceutical services tend to attract more users, thereby increasing service output and reducing underutilization of resources-one of the major sources of inefficiency in health systems [59].

The existence of a pharmacy may reflect broader organizational and managerial capacities within health facilities. Pharmaceutical management requires functional systems for procurement, storage, stock monitoring, and distribution. As such, facilities with pharmacies are likely to exhibit stronger governance and better management practices overall. Evidence suggests that managerial capacity is a significant determinant of health facility performance, including efficiency outcomes [29].

However, this association should be interpreted with caution. The availability of a pharmacy may also serve as a proxy for better-resourced facilities, which are more likely to have adequate infrastructure, qualified staff, and stronger integration within the health system. Previous studies have highlighted that structural factors such as infrastructure, human resources, and equipment availability are closely linked to efficiency levels [29, 30, 60]. Therefore, the observed effect may partially reflect these underlying advantages rather than the independent contribution of pharmacy availability alone.

The findings also revealed a negative association between the proportion of adolescents in a facility’s coverage area and technical efficiency scores for ASRH services. An increase in the adolescent proportion correlates with a reduced likelihood of highly technical efficiency scores. This may be attributed to elevated service demand: facilities serving a larger adolescent population often face heightened consultation volumes, and, in the absence of adequate human resources and effective management, may struggle to deploy available resources efficiently [61, 62].

### Policy implications

*Strengthen the integration of mental health services within primary health care*. The extremely low availability of mental health services in health facilities in the Maradi and Niamey regions highlights the urgent need for stronger policy commitment to mental health integration within primary health care. The government should prioritize the implementation of national mental health strategies that expand service provision at the primary care level, including training of frontline health workers, integration of mental health screening into routine services, and improved referral systems. This would help reduce the reliance on traditional medicine as the primary source of mental health care and expand access to evidence-based services.

*Improve the efficiency of ASRH services*. Although a high proportion of health facilities offer ASRH services, the low average technical efficiency score (45%) indicates substantial inefficiencies in resource utilization. Health sector policies should therefore focus not only on expanding service coverage but also on improving the efficiency of service delivery. This could involve enhancing facility management, refining resource allocation methods, and introducing performance monitoring systems to ensure existing resources achieve the best possible health outcomes.

*Expand training and capacity building for ASRH service providers.* The findings show that facilities with trained ASRH providers demonstrate significantly higher technical efficiency. Policymakers should therefore scale up training programs on adolescent-friendly service provision for health workers. Continuous professional development and specialized training could improve service quality, strengthen adolescents’ trust in health facilities, and ultimately increase service utilization and efficiency.

*Strengthen human resources and service capacity in high-demand areas*. The negative relationship between the proportion of adolescents in a facility’s catchment population and technical efficiency suggests that high demand may strain existing resources. Policies should therefore prioritize the allocation of additional staff, equipment, and operational resources to facilities serving large adolescent populations. Such targeted resource allocation could help facilities better manage service demand and improve efficiency.

*Promote equitable and gender-sensitive leadership in health facility management.* Differences in efficiency associated with the gender of facility heads indicate that leadership and management capacity may influence service performance. Policymakers should invest in leadership and management training for health facility managers while ensuring gender equity in leadership opportunities. Strengthening managerial capacity can enhance organizational performance and resource utilization.

*Strengthening pharmaceutical systems*—particularly ensuring continuous availability of essential medicines—could be an effective strategy to improve health facility efficiency. Interventions aimed at improving supply chain management, enhancing facility autonomy, and reducing stock outs may lead to substantial gains in performance in resource-limited settings.

### Strengths and limitations

This research is among the first to investigate both mental health and adolescent sexual and reproductive health services offered in Niger. It uniquely addresses resource use in health facilities from a technical efficiency angle. The findings contribute to ongoing political and scientific discussions surrounding these issues in developing countries, while also supplying stakeholders with valuable evidence for shaping programs and policies that support better adolescent mental health and sexual and reproductive health outcomes.

However, several limitations should be considered when interpreting the findings of this study. First, potential selection bias may arise from the exclusion of 55 eligible facilities. Excluding observations because of facility ineligibility and missing data may introduce systematic bias and inflate estimates of technical efficiency [63]. With fewer observations, the efficiency frontier is constructed from a smaller set of cases, which may make remaining units appear more efficient than they are. Nonetheless, excluding facilities that did not provide adolescent sexual and reproductive health services helps limit overestimation, and random exclusion due to missing data may reduce the risk of systematic selection bias.

Second, the analysis lacks direct measures of care quality. Although we examine structural and operational characteristics associated with outcomes, we do not include clinical process indicators such as adherence to treatment protocols, patient experience measures, or adverse event rates. Unmeasured differences in quality across facilities may therefore confound or partially explain the observed associations. As a result, it is not possible to determine whether higher staffing levels, for example, lead to better clinical outcomes or simply correlate with other quality-enhancing factors.

Third, this study uses a cross-sectional design and relies partly on self-reported data, which may be subject to desirability or reporting bias. Because the data capture facility characteristics and outcomes at a single point in time, the analysis can identify associations but cannot establish causal direction, account for temporal change, or exclude reverse causality. For example, facilities with better outcomes may attract greater resources, such as staffing or equipment, rather than these resources necessarily causing improved outcomes. Longitudinal research is needed to clarify temporal ordering and examine changes within facilities over time.

Fourth, the pseudo-R^2^ value for the fractional regression is 0.048, indicating that the included predictors explain only a limited proportion of the variance in the outcome. Although low explanatory power is common in health services research involving nonlinear models and complex outcomes, this result suggests that substantial variation remains unexplained by the covariates included in the analysis. Important factors such as leadership quality, organizational culture, socioeconomic characteristics of the catchment population, and unmeasured case mix may therefore have been omitted. Accordingly, model-based predictions should be interpreted with caution, and future research should incorporate a broader range of explanatory variables. Nevertheless, several authors have noted that pseudo-R^2^ values in nonlinear models, including fractional response regressions, are not directly comparable to R^2^ values from linear regression models and are typically lower even when the model specification is appropriate [41, 64]. Methodological guidance therefore recommends focusing on the direction, magnitude, and statistical significance of coefficients and marginal effects rather than overall model fit [41, 64]. In this context, the findings should be interpreted as exploratory and requiring further investigation.

Finally, the ratio of observations to parameters was 7, whereas a minimum ratio of 10 is generally recommended for robust estimation in frontier analysis. To mitigate this concern, we assessed the stability and plausibility of estimated coefficients and focused interpretation on overall efficiency scores and patterns rather than individual parameter estimates [65, 66].

## Conclusion

The limited research available on adolescent mental health and the lack of resources to provide sexual and reproductive health services for adolescents formed the focus and basis of this paper. Beyond the aim of producing evidence-based recommendations for action, the findings suggest that the issue of mental health remains neglected in Niger. The efficient use of available resources by primary health care facilities also appears problematic. The results showed that very few primary health care facilities offered mental health services and that these facilities could increase their capacity to use available resources efficiently to provide more sexual and reproductive health services. The policy implications analysed can serve as a foundation for stakeholders to better define courses of action for improving the mental, sexual and reproductive health of adolescents. The limitations identified implies that the findings should be considered as exploratory and requiring further research on the same issue.

## Electronic supplementary material

Below is the link to the electronic supplementary material.

Supplementary Material 1

Supplementary Material 2

## Data Availability

The data used are primary data collected specifically for the purposes of the study. The data can be accessed by sending a request to the first author of this paper.

## Abbreviations

Adowa: Adolescent Health West Africa
AE: Allocative Efficiency
AFD: French Development Agency
AMH: Adolescent Mental Health
ASRH: Adolescent and Sexual and Reproductive Health
ASRH: Adolescent sexual and Reproductive
DEA: Data Envelopment Analysis
EE: Economic efficiency
FDA: French Development Agency
FRM: Fractional Response Model
GLM: Generalized linear models
HIV: Human Immunodeficiency Virus
ICPD: International Conference on Population and Development
IPPF: Pathfinder and the International Planned Parenthood Federation
IPPF: International Planned Parenthood Federation
LR: likelihood ratio
MRC: Medical Research Council
OLS: Ordinary Least Squares
QMLE: Quasi-Maximum Likelihood Estimation
RNG: Random Number Generator
SFA: Stochastic Frontier Analysis
SFA: Stochastic Frontier Analysis
SHR: Sexual and Reproductive Health
STIs: Sexually Transmitted Infections
TE: Technical Efficiency
UK: United Kingdom
UNFPA: United Nations Fund for Population
UNICEF: United Nations International Children’s Emergency Fund
WHO: World Health Organisation
