# Supplementary material for: Availability of adolescent mental, sexual and reproductive health services (ASRH) and technical efficiency of ASRH service delivery in Niger
Source: BMC Health Serv Res. 2026 Jun 30;26:1282. doi: 10.1186/s12913-026-15014-y (PMC13584480; doi:10.1186/s12913-026-15014-y)
Supplement: Supplementary file 1 [file 12913_2026_15014_MOESM2_ESM.pdf]

LASDEL
Laboratoire d'Etudes et de Recherches sur les Dynamiques Sociales et le Développement Local

Priorities for the implementation of policies, programmes and primary care relating to mental, sexual and reproductive health and the well-being of adolescents in West Africa

Questionnaire for providers in district hospitals, health centres and clinics.

**Questionnaire number :**				

A: Free and informed consent form
NOTE: to be given to the person in charge of the healthcare facility

Hello, my name is .... I am working on the ADOWA project coordinated by the Laboratoire de études et de recherche sur les dynamiques sociales et le développement local (Lasdel). Despite the implicit acceptance of a more holistic approach to adolescent health and well-being, and the inclusion of mental health as a priority issue for adolescents in the ECOWAS sub-region, there appear to be gaps in information about the funding, effectiveness and comprehensiveness of adolescent health and well-being interventions. This study is being conducted in three West African countries (Ghana, Niger and Burkina Faso). In each selected region, a district hospital and a number of health centres/clinics have been chosen to enable an assessment of the effectiveness of health structures in providing specific services to adolescents. I would like to ask you a few questions about your health facility. The questions generally concern the general conditions of your facility and the key processes relating to the adolescent services you provide. I would like to assure you that the information you provide will remain strictly confidential. Under no circumstances will your identity be revealed to anyone outside the research team. Your participation in this work is very important to help us gather relevant information for our studies and contribute to improving the delivery of adolescent health care in Niger.

You are free to take part in this study, which will take between 30 and 45 minutes of your time. If you agree to take part in this study, you may skip certain questions if they do not suit you. You may also interrupt the interview if necessary. You can also ask any questions you may have about the study at this stage. You can also ask your colleagues to answer any questions that only they are able to answer.

Do you agree to take part in this study on the basis of the information I have given you?

YES NO ◯ ◯

WHETHER THE INTERVIEWEE AGREES TO BE INTERVIEWED

....
NAME Signature Date

Start time of interview		For example (15:40 h)
End time of interview		For example (5pm)

IDENTIFICATION OF THE STRUCTURE			
			Code
Region: 4. Maradi 8. Niamey			
Health district:			
Name of establishment :			
GPS coordinates:			
Type of structure/institution		Integrated health centre (IHC)	1
		Health centre	2
		Clinic	3
		Treatment room	4
		District hospital	5
		Health district	6
		ANBEF Clinic	
		Other, please specify................................	7
Location of the structure/establishment		Urban	1
		Rural	2
Managing Authority		Government	1
		Mission (Christian/Islamic)	2
		Private	3
		Private-Government (Mixed)	4
		Other, please specify............................	5
**RESPONDENT INFORMATION**			
This section collects information about the respondent (i.e. the characteristics of the person in charge of the structure or a person designated by him/her).			
1	What is the job title of the Head of School?	Medical consultant (including doctors with specialist training)	1
		Hospital administrator	2
		Medical assistant	3
		Public health nurse	4
		Midwife	5
		Registered nurse (including nurses with specialist training)	6
		Assistant midwife	7
		Auxiliary nurse	8
		Community health workers	9
		Other (specify).......................	10
2	How long have you worked in this position?	☐ ☐ ☐ Year month day	
3	Like the person in charge of the establishment?	Men	1
		Woman	2
**Section A: General information about the health care organisation**			
4	Population in your coverage area [What is the percentage of teenagers (aged 10-19)?		
5	Coverage radius (in kilometres of greatest distance)		
6	Number of vehicles on the premises		
7	Number of motorbikes in operation at the facility		
8	Number of racing bikes in the establishment		

9	Does your establishment have the following functional equipment (......)? (Inventory of equipment°)		
9a1	Electricity supply via the national grid	Yes	1
		No	2
9a2	Average number of hours of electricity per day?		
9b1	Electricity generator	Yes	1
		No	2
9b2	Does it start as soon as the power is cut?		
9c1	Solar energy system	Yes	1
		No	2
9c2	Does it supply the whole installation or just part of it?		
9c3	Which device does it power?		
10	Number of consultation rooms in your establishment		
10a	Number of consultation rooms in your establishment reserved for adolescents		
11	Number of spaces reserved for teenagers		
12	Number of operating theatres **(if none, write 0)**		
13	Number of fridges/freezers in operation **(if none, enter 0)**		
14	Number of functional beds **(Indicate separate beds for teenagers if available)**		
	Recovery room beds		
	Consultation room beds		
	Delivery room beds		
	Inpatient beds (observation beds)		
15	I'd like to ask you what the usual opening hours are at this establishment. Enter the time in 24-hour units (for example, 0900, 1430).		
	Time	Working hours	
15a	Days of the week		
15b	Saturdays		
15c	Sundays		
15d	Public holidays		
16	What is the facility's main source of water?	Pump water/Cock	1
		Water truck and water supplier services	2
		Drilling	3
		Protected well	4
		Unprotected well	5
		Excavation/pond/barrage	6
		Other (specify).............................	7
17	Does this establishment have a regular water supply?	Yes	1
		No	2
18	Does the establishment have a working official telephone (landline or mobile)?	Yes	1
		No	2
19	What is the telephone number?		
20	Does the establishment have a computer in working order to compile data/statistics for the departments?	Yes	1
		No	2

Section B: General information (services provided, trends)					
		Year			
		In 2018	In 2019	In 2020	In 2021
21	Total number of outpatients				
22	Number of adolescents (aged 10-14) who have received sexual and reproductive health advice				
23	Number of adolescents (15-19 years) who have received a sexual and reproductive health consultation				
24	Number of adolescents (aged 10-14) who received a mental health consultation				
25	Number of adolescents (15-19 years) who received a mental health consultation				
26	Total number of patients hospitalised **(if none, enter 0)**				
27	Number of adolescents (aged 10-14) hospitalised for sexual and reproductive health care				
28	Number of adolescents (15-19 years) hospitalised for sexual and reproductive health care				
29	Number of adolescents (aged 10-14) hospitalised for mental health treatment				
30	Number of adolescents (15-19 years) hospitalised for mental health care				
31	Number of functional beds				
31a	Number of beds in recovery rooms (convalescence)				
31b	Number of recovery room beds (for teenagers)				
31c	Number of consultation room beds				
31d	Number of consultation room beds (for adolescents)				
31e	Number of beds for delivery rooms				
31f	Number of beds for delivery rooms (for teenagers)				
31g	Number of inpatient beds (observation beds)				
31h	Number of inpatient beds (observation beds) (for adolescents)				
32	Number of observation bed days for hospitalised adolescents (if none, enter 0)				
33	Number of adolescent patients hospitalised (10-14 and 15-19)				
34	Average length of stay for adolescents hospitalised **(if none, enter 0)**				

Section C: HEALTH SERVICES FOR YOUNG PEOPLE IN THE INSTITUTION			
**Section C1: SEXUAL AND REPRODUCTIVE HEALTH SERVICES**			
34		Yes	1

	Does this establishment offer sexual and reproductive health services for adolescents?	No	2
35	Please check the services offered by this establishment	Prenatal care	1
		Birth	2
		Postnatal care	3
		Complete abortion care (manual vacuum aspiration)	4
		Full care in the event of a complete abortion (medical abortion)	5
		Family planning	6
		Other, please specify....................	7
36	Do you have the national guidelines for the provision of sexual and reproductive services to adolescents available in this establishment today?	Yes	1
		No	2
37	If you answered yes to question [36], is a copy available for consultation?	Yes	1
		No	2
38	Do you have any providers of sexual and reproductive health services for adolescents who have received training in the provision of health services for adolescents in the last two years?	Yes	1
		No	2
39	If you answered yes to question [38], how many people have been trained in the last two years?		
**Section C2: MENTAL HEALTH SERVICES**			
40	Does this facility offer mental health services for adolescents?	Yes	1
		No	2
41	Please indicate the psychiatric illnesses for which the provider is responsible.	Alcohol and substance use disorders	1
		Depression	2
		Anxiety disorders	3
		Attempted suicide and self-mutilation	4
		Psychotic disorders	5
		Other, please specify....................	6
42	Which of the following services are available in your institution for the provision of mental health services to adolescents?	Basic psychosocial counselling and support services	1
		Laboratory screening for substance use	2
		First-line oral psychoactive drugs, such as Prozac.	3
		Emergency services, for example in cases of acute psychosis or attempted suicide.	4
43	Do you now have national guidelines for the provision of mental health services to adolescents?	Yes	1
		No	2
44	If you answered yes to question [43], is a copy available for consultation?	Yes	1
		No	2
45	Do you have any providers of adolescent mental health services who have received training in the provision of adolescent mental health services in the last two years?	Yes	1
		No	2
46	If you answered yes to question [45], how many people have been trained in the last two years?		
47	Is there anything that could be done to improve your adolescent health services?	Yes	1
		No	2

Section D: ESTABLISHMENT STAFF		2018	2019	2020	2021
49	Number of doctors (full-time) in your establishment (in post and on annual leave)				
50	Number of part-time doctors (locums)				
51	Number of medical assistants in your establishment (in post and on annual leave)				
52	Number of part-time medical assistants (substitutes)				
53	Number of midwives in your establishment (including midwives in training)				
54	Number of part-time midwives (locums)				
55	Number of nurses in your establishment (including nurses in training)				
56	Number of part-time nurses (substitutes)				
57	Number of medical technicians in your establishment (radiography, laboratory, field technician, etc.)				
58	Number of part-time medical technicians (substitutes)				
59	Number of employees trained in adolescent sexual and reproductive health				
60	Number of administrative staff in your establishment				

Section D1: PROFESSIONAL STAFF PROVIDING SERVICES TO TEENAGERS (FOR THE MOST RECENT YEAR)

61 Which doctors work directly with teenagers on this site? **List them all, regardless of who pays them.**

Doctor	Specialisation, for example, Obstetrics. And gynaecology (O&G)	Part-time/full-time	Received training in caring for adolescents (in the past year?)	Total number of hours worked per week	Number of hours per week devoted to the programme/service for adolescents
A					
B					
C					
D					
E					

62 Which midwives on this site work directly with teenagers? **List them all, regardless of the organisation that pays them.**

Midwives	Part-time/full-time	Received training in caring for adolescents (in the past year?)	Total number of hours worked per week	Number of hours per week devoted to the adolescent programme/service
A				
B				
C				
D				
E				

63	Which nurses work directly with teenagers on this site? **List them all, regardless of the organisation that pays them**.				
	Nurses	Part-time/full-time	Received training in caring for adolescents (in the past year?)	Total number of hours worked per week	Number of hours per week devoted to the adolescent programme/service
	A				
	B				
	C				
	D				
	E				
64	Which medical assistants work directly with teenagers on this site? **List them all, regardless of the organisation that pays them.**				
	Assistant Doctors	Part-time/full-time	Received training in caring for adolescents (in the past year?)	Total number of hours worked per week	Number of hours per week devoted to the adolescent programme/service
	A				
	B				
	C				
	D				
	E				
65	Which medical technicians (x-ray, laboratory, field technician) work directly with the adolescents on this site? **List them all, regardless of who pays them.**				
	Medical technician/Grade	Part-time/full-time	Received training in caring for adolescents (in the past year?)	Total number of hours worked per week	Number of hours per week devoted to the adolescent programme/service
	A				
	B				
	C				
	D				
	E				

Section D2: Administrative and other support staff

66	What administrative or other support staff at this site provide administrative and other support to the adolescent health unit? **List them all, regardless of who pays them. (General administration if adolescent units are not available)**				
	ADMINISTRATIVE STAFF/POSITION	Part-time/full-time	Received training in caring for adolescents (in the past year?)	Total number of hours worked per week	Number of hours per week devoted to the adolescent programme/service
	A				
	B				
	C				
	D				
	E				

Section E: MEDICAL SUPPLIES AND CONSUMABLES

67	Do you have a functional pharmacy/dispensary that meets the medical needs of adolescents? [A functional pharmacy must have medicines (abortion kit, contraceptive pills), medical supplies (condoms, IUDs, manual suction)]. A pharmacy must have a list of basic medicines needed for adolescents' mental health care: antidepressants (fluoxetine), oral antipsychotics (risperidone), parenteral psychoactive medicines for emergency care (IM chlorpromazine, IV diazepam).	Yes	1
		No	2

68 Describe each of the medical consumables and other supplies you use for adolescents each year.

	2019			2020			2021		
Type of medical supply	Quantity used	Number of adolescents {10-14 years} receiving this type of medical supply	Number of adolescents {15-19} receiving this type of medical supply	Quantity used	Number of adolescents {10-14 years} receiving this type of medical supply	Number of adolescents {15-19} receiving this type of medical supply	Quantity used	Number of adolescents {10-14 years} receiving this type of medical supply	Number of adolescents {15-19} receiving this type of medical supply
Contraceptive pills									
Condoms									
Misoprostol or Cytotec									
Manual vacuum suction kit									
Emergency box for childbirth (eclampsia kit)									
Emergency box for an ectopic pregnancy									
Medicines for STIs									
Medication for candidiasis									
Medicines for anaemia									

Section E1: LABORATORY TESTS

69	Do you have a functional medical laboratory that meets the medical needs of adolescents? [A functional medical laboratory for teenagers would include pregnancy tests and tests for sexually transmitted infections (gonorrhoea, AIDS, Chlamydia)].	Yes	1
		No	2

70 Describe each of the laboratory tests associated with care per year for adolescents [2019-2021]. **The list includes pregnancy tests, gonorrhoea, chlamydia, HIV tests.**

	2019			2020			2021		
Laboratory test (specify by type: RDT or laboratory; sexual and reproductive health or mental health)	Number of tests	Number of adolescents {10-14 years} receiving this type of laboratory test	Number of adolescents {15-19} receiving this type of laboratory test	Number of tests	Number of adolescents {10-14 years} receiving this type of laboratory test	Number of adolescents {15-19} receiving this type of laboratory test	Number of tests	Number of adolescents {10-14 years} receiving this type of laboratory test	Number of adolescents {15-19} receiving this type of laboratory test
Pregnancy tests (RDT, rapid diagnostic test)									
Pregnancy tests (laboratory)									
STI test for HIV (Syphilis, AIDS, Chlamydia, Gonorrhoea)									
Urine screening for psychoactive substances									

**Section F: FINANCING SOURCES**						
71	Describe each source of funding associated with the specific activity for adolescents by year					
	Source of financing	Year	Total amount received for all services	Total devoted to ASRH services	Amount spent	Comments
	Central/local government funding	2019				
		2020				
		2021				
	Funding of national institutions, associations and NGOs	2019				
		2020				
		2021				
	Funding for institutions/missions/national NGOs	2019				
		2020				
		2021				
	Financing through user fees	2019				
		2020				
		2021				
	Funding from other sources. Please specify..................	2019				
		2020				
		2021				

**QUESTIONS FOR THE INTERVIEWER**				
**TO BE COMPLETED BY THE INTERVIEWER SHORTLY AFTER THE INTERVIEW**				
			Code	Comment
1	How many times did you visit this establishment to complete this questionnaire?			
2	How many people responded to this questionnaire?			
2a	Indicate their name/position			

**Conditions for offering services within the establishment**			
1	Describe the general cleanliness of the waiting rooms.	Clean and tidy appearance	1
		Slightly dirty appearance, with minor spills, debris or rubbish present.	2
		Very dirty appearance, with heavy spills, debris or rubbish on chairs or floors.	3
		Not applicable, no waiting room	4
		Missing/unobserved data	5

2	Describe the general cleanliness of the examination room(s).	Clean and tidy appearance	1
		Slightly dirty appearance, with minor spills, debris or rubbish present.	2
		Very dirty appearance, with heavy spills, debris or rubbish on chairs or floors.	3
		Not applicable, no waiting room	4
		Missing/unobserved data	5
3	Describe the general setting of the examination or consultation room(s).	Room with visual and auditory privacy	1
		Room with visual privacy	2
		Room with auditory privacy only	3
		Room without privacy	4
		Not applicable, no examination room	5
		Missing/unobserved data	6

The questionnaire has been approved for data entry	
**SIGNATURE**	**SIGNATURE**
SUPERVISOR'S NAME	NAME OF INTERVIEWER

Thank you for your participation!
