# Supplementary material for: Availability of adolescent mental, sexual and reproductive health services (ASRH) and technical efficiency of ASRH service delivery in Niger
Source: BMC Health Serv Res. 2026 Jun 30;26:1282. doi: 10.1186/s12913-026-15014-y (PMC13584480; doi:10.1186/s12913-026-15014-y)
Supplement: Supplementary file 2 [file 12913_2026_15014_MOESM1_ESM.docx]

**Appendix**

**Appendix 1:** Characteristics of healthcare facilities included and excluded from the various analyses

| **Characteristics** | **Healthcare facilities not providing SSRA services** | **Healthcare facilities providing SSRA services not included in the efficiency analysis due to a lack of information on inputs or outputs** | **Healthcare facilities included in the efficiency analysis** | **Total** |  |
| --- | --- | --- | --- | --- | --- |
|  |  |  |  |  |  |
| **Place of residence** | | | | |  |
| *Urbain* | 13 | 27 | 32 | 72 |  |
| *Rural* | 5 | 10 | 73 | 88 |  |
| *Total* | 18 | 37 | 105 | 160 |  |
| **Type of Health facility** | | | | |  |
| *Integrated health center* | 15 | 37 | 103 | 155 |  |
| *Clinic* | 3 | 0 | 2 | 5 |  |
| *Total* | 18 | 37 | 105 | 160 |  |
| **Gender of health facility head** | | | | |  |
| *Man* | 9 | 29 | 26 | 64 |  |
| *Woman* | 9 | 8 | 79 | 96 |  |
| *Total* | 18 | 37 | 105 | 160 |  |
| **Region** | | | | |  |
| *Maradi* | 6 | 7 | 87 | 100 |  |
| *Niamey* | 12 | 30 | 18 | 60 |  |
| *Total* | 18 | 37 | 105 | 160 |  |
| **Management autority** | | | | |  |
| Private | 3 | 2 | 7 | 12 |  |
| Government | 15 | 35 | 98 | 148 |  |
| Total | 18 | 37 | 105 | 160 |  |

**Appendix 2:** Test result Model with Translog function vs Model with Cobb-Douglas function

**Lrtest**

$\begin{matrix} Ho:\beta_{ij}=0 \\ Ha:\beta_{ij}\neq0 \end{matrix}$ Cobb douglas vs Translog

**sfcross lnNmbrConsSSRA X1 X2 X3 X4, model (bc) distribution(exp) ort (o)**

**sfcross lnNmbrConsSSRA X1 X2 X3 X4 X1² X2² X3² X4² X1X2 X1X3 X1X4 X2X3 X2X4 X3X4, model (bc) distribution(exp) ort (o)**

est store M2

We cannot accept H0. Translog is better than Cobb-douglas
